# Synthesis of 8-Aryl-*O*-methylcyanidins and Their Usage for Dye-Sensitized Solar Cell Devices

**DOI:** 10.3390/ijms18020427

**Published:** 2017-02-16

**Authors:** Yuki Kimura, Kin-ichi Oyama, Yasujiro Murata, Atsushi Wakamiya, Kumi Yoshida

**Affiliations:** 1 Graduate School of Information Science, Nagoya University, Chikusa, Nagoya 464-8601, Japan; kimura.yuki@i.mbox.nagoya-u.ac.jp; 2 Chemical Instrumentation Facility, Research Center for Materials Science, Nagoya University, Chikusa, Nagoya 464-8602, Japan; oyama@cic.nagoya-u.ac.jp; 3 Institute for Chemical Research, Kyoto University, Gokasho, Uji, Kyoto 611-0011, Japan; yasujiro@scl.kyoto-u.ac.jp (Y.M.); wakamiya@scl.kyoto-u.ac.jp (A.W.)

**Keywords:** 8-aryl-*O*-methylcyanidin, 8-aryl-*O*-methylquercetin, dye-sensitized solar cells, *O*-methylcyanidin, *O*-methylquercetin

## Abstract

Anthocyanins as natural pigments are colorful and environmentally compatible dyes for dye-sensitized solar cells (DSSCs). To increase the efficiency, we designed and synthesized unnatural *O*-methylflavonols and *O*-methylcyanidins that possess an aryl group at the 8-position. We synthesized *per*-*O*-methylquercetin from quercetin, then using selective demethylation prepared various *O*-methylquercetins. Using the Suzuki-Miyaura coupling reaction, 8-arylation of *per*-*O*-methylquercetin was achieved. Using a LiAlH_4_ reduction or Clemmensen reduction, these flavonols were transformed to the corresponding cyanidin derivatives in satisfactory yields. Using these dyes, we fabricated DSSCs, and their efficiency was investigated. The efficiency of *tetra*-*O*-methylflavonol was 0.31%. However, the introduction of the 8-aryl residue increased the efficiency to 1.04%. In comparison to these flavonols, *O*-methylcyanidins exhibited a lower efficiency of 0.05% to 0.52%. The introduction of the 8-aryl group into the cyanidin derivatives did not result in a remarkable increase in the efficiency. These phenomena may be due to the poor fit of the HOMO-LUMO level of the dyes to the TiO_2_ conduction band.

## 1. Introduction

Anthocyanins are natural pigments that are widespread in higher plants, and the current number of isolated pigments are approaching one thousand [1,2,3,4,5]. The distinct characteristics of anthocyanins include their beautiful colors, with a wide variety of colors ranging from red to purple and blue in nature [1,5,6]. In addition, the color changes based on the pH of the media and the existence of co-pigments and/or metal ions [1,2,3,4,5,6]. Due to these properties, anthocyanins have been used as safe food colorants for a long time [7,8]. Recently, anthocyanins have attracted increasing attention as functional colorants for lifestyle-related diseases [9,10,11] and potential dyes for dye-sensitized solar cells (DSSCs) devices [12,13], which are next-generation solar cells with low production costs and flexibility. To expand these studies, development of an efficient synthetic procedure of dyes is essential.

To synthesize anthocyanins, the metal reduction of flavonols to their corresponding anthocyanidins (anthocyanins) has been previously reported in the early 20th century [14,15,16]. This synthetic route is very effective because the transformation is achieved in a single-step reaction without any protecting groups at the polyphenolic hydroxyl groups. A long time after those reports, Brouillard and his group reported the reduction of flavonols using Zn–Hg in 1995 [17]; however, the yield was not satisfactory. We re-investigated this reaction procedure and improved the reaction conditions using Zn powder rather than Zn amalgam and reported that anthocyanins can be obtained in a yield of more than 80% [18]. Using this method, several anthocyanins including acylated anthocyanins can be obtained from the corresponding flavonols [18,19]. This method was expanded to synthesize unnatural anthocyanidins. For this purpose, we first synthesized various methylated quercetins [20] and 8-substituted quercetins [21]. After selective demethylation, the reduction of these flavonols afforded substituted cyanidins in good yields.

DSSCs studies using anthocyanins have been carried out by several groups [22,23,24,25,26] because anthocyanin contains no metal ions and is environmentally friendly. However, the efficiency *η*% value of the DSSCs using pure anthocyanins and/or extracts from plants was low (i.e., approximately 1.0%) [22,23,24,25]. In 2013, Calogero et al. reported an efficiency of 2.2% using synthetic anthocyanidin [27]. We fabricated DSSCs using five pure natural anthocyanidin 3-*O*-glucosides with different chromophores and compared the color of the cells as well as their efficiencies [28]. These pigments resulted in beautiful cells that were blue due to petunidin 3-*O*-glucoside and purplish blue due to delphinidin 3-*O*-glucoside. However, the efficiency was still not high (i.e., approximately 1% to 1.4%) [28]. The time-dependent density functional theory (TD-DFT) calculations for the anthocyanidin dye model cluster systems (dye/(TiO_2_)_38_) revealed that both indirect (Type-I) and direct (Type-II) electron injection mechanisms may co-exist [28]. In addition, the highest occupied molecular orbital (HOMO)-the lowest unoccupied molecular orbital (LUMO) level of the dye may not match well with the conduction band of TiO_2_, which may result in low efficiency of the anthocyanin dye-based DSSCs. A preliminary calculation indicated that introduction of an aryl group at the 8-position may increase the HOMO level of the dye to match the conduction band of TiO_2_. Therefore, we designed 8-arylanthocyanidin for use as a DSSC dye. We synthesized *O*-methylquercetins and 8-aryl-*O*-methylquercetins from quercetin. The number of methyl moieties was varied by selective demethylation [20]. These methylquercetin derivatives were transformed to the corresponding *O*-methylcyanidins and 8-aryl-*O*-methylcyanidins via reduction. Using the synthesized derivatives, we fabricated DSSCs, and the photoelectric conversion efficiency was measured.

## 2. Results and Discussion

### 2.1. Synthesis of O-Methylquercetins

**Scheme 1:**
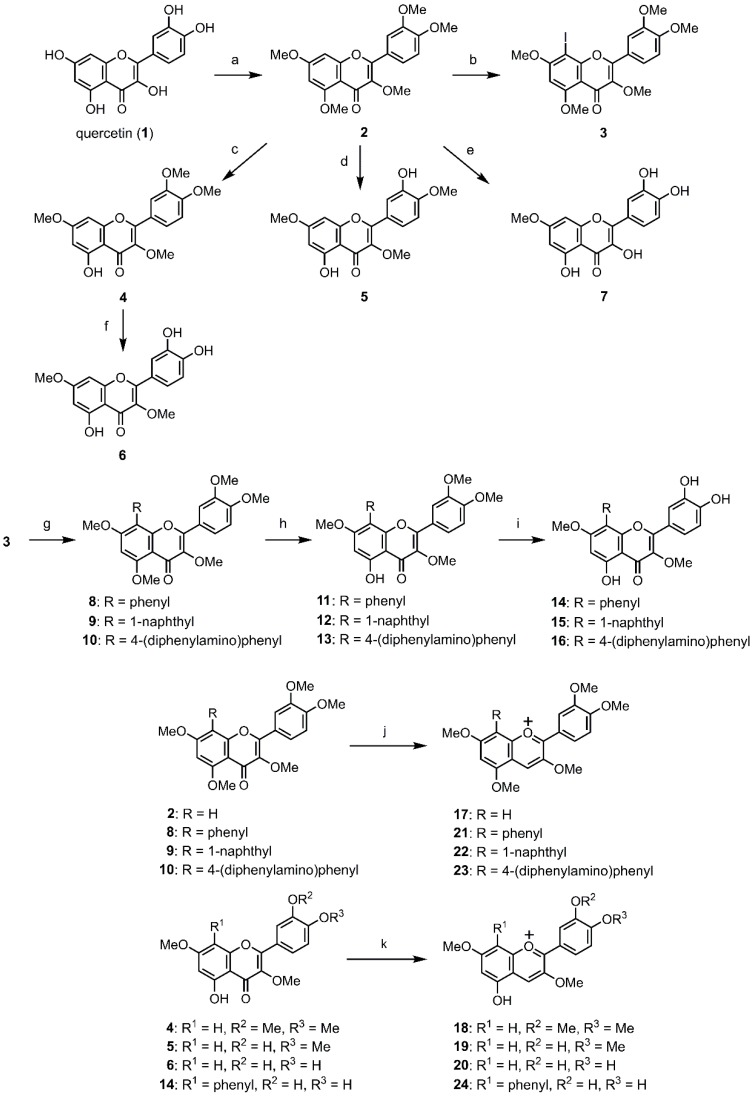
Synthetic plan of *O*-methylquercetins (**2**–**16**) and *O*-methylanthocyanidins (**17**–**23**). (**a**) MeI (29 eq.), NaH (7.6 eq.), DMF, 80 °C; (**b**) NIS (1.2 eq.), DMF, 70 °C; (**c**) BBr_3_ (2.0 eq.), MeCN, rt; (**d**) BBr_3_ (2.0 eq.), CH_2_Cl_2_, −30 °C; (**e**) BBr_3_ (3.0 eq.), CH_2_Cl_2_, 0 °C; (**f**) BCl_3_ (5.3 eq.), TBAI (5.3 eq.), CH_2_Cl_2_, −20 °C; (**g**) **8**: phenylboronic acid (2.0 eq.), Pd(OAc)_2_ (3.0 mol %), K_3_PO_4_ (3.0 eq.), toluene, 100 °C; **9**: 1-naphthalenebronic acid (2.0 eq.), Pd(OAc)_2_ (3.0 mol %), K_3_PO_4_ (3.0 eq.), PPh_3_ (3.0 mol %), CPME, 100 °C; **10**: 4-(diphenylamino)phenylboronic acid (1.5 eq.), Pd(OAc)_2_ (1.5 mol %), K_2_CO_3_ (2.0 eq.), EtOH/H_2_O, 80 °C; (**h**) BBr_3_ (3.0 eq.), CPME, 0 °C → rt; (**i**) BCl_3_ (5.3 eq.), TBAI (5.3 eq.), CH_2_Cl_2_, −20 °C; (**j**) LiAlH_4_ (4.0 eq.), THF, rt; (**k**) Zn, HCl-MeOH, 0 °C and then air, HCl-MeOH, rt.

The synthetic strategy for various *O*-methylquercetins and *O*-methylcyanidins bearing an 8-aryl group is shown in Scheme 1. First, we prepare *per*-*O*-methylquercetin (**2**) via permethylation of quercetin (**1**), and then, various *O*-methylquercetins with different numbers of methyl moieties were prepared via regioselective demethylation. In addition, regioselective iodination afforded 8-iodo *per*-*O*-methylquercetin (**3**), and then, Suzuki-Miyaura coupling afforded 8-aryl-*O*-methylquercetins. The quercetin derivatives were reduced to yield the corresponding *O*-methylcyanidins. Using this scheme, quercetin and cyanidin derivatives with different numbers of *O*-methyl residues and varied 8-aryl groups were prepared.

3,5,7,3′,4′-*penta*-*O*-methylquercetin (**2**) was prepared in 86% yield by permethylation of **1** using MeI and NaH in dimethylformamide (DMF) [20]. Based on our regioselective demethylation method via *per*-*O*-methylquercetin (**2**), 3,7,3′,4′-*tetra*-*O*-methylquercetin (**4**), 3,7,4′-*tri*-*O*-methylquercetin (**5**), 3,7-*di*-*O*-methylquercetin (**6**), and 7-*O*-methylquercetin (**7**) were synthesized [20]. Demethylation of **2** with BBr_3_ in MeCN at room temperature afforded 3,7,3′,4′-*tetra*-*O*-methylquercetin (**4**) as the sole product in 98% yield due to neighboring group participation by the 4-carbonyl group. The regioselective demethylation to synthesize 3,7,4′-*tri*-*O*-methylquercetin (**5**) and 7-*O*-methylquercetin (**7**) was achieved by controlling the amount of BBr_3_ as well as the reaction temperature. **5** was synthesized in 40% yield using 2.0 eq. of BBr_3_ in CH_2_Cl_2_ at −30 °C. In addition, **7** was prepared in 25% yield using 3.0 eq. of BBr_3_ in CH_2_Cl_2_ at 0 °C. 3,7-*di*-*O*-methylquercetin (**6**) was obtained in 83% yield from **4** using a combination of BCl_3_ and *tetra*-butylammonium iodide (TBAI) in CH_2_Cl_2_.

8-Iodo-*per*-*O*-methylquercetin (**3**) was prepared in 90% yield from **2** using *N*-iodosuccinimide (NIS) at room temperature (rt) [21,29]. The Suzuki-Miyaura coupling reaction [30,31,32] of **3** with phenylboronic acid in the presence of Pd(OAc)_2_ and K_3_PO_4_ in toluene afforded 3,5,7,3′,4′-*penta*-*O*-methyl-8-phenylquercetin (**8**) in 90% yield [21]. For the coupling reaction of **3** with 1-naphthaleneboronic acid, the PPh_3_ ligand was required. The reaction in the presence of Pd(OAc)_2_, PPh_3_, and K_3_PO_4_ in cyclopentyl methyl ether (CPME) afforded 3,5,7,3′,4′-*penta*-*O*-methyl-8-(1-naphthyl)quercetin (**9**) in 69% yield [21]. The coupling reaction between **3** and 4-(diphenylamino)phenylboronic acid was conducted according to Liu’s ligand-free conditions [33]. The reaction in the presence of Pd(OAc)_2_, and K_3_CO_3_ in EtOH/H_2_O afforded 3,5,7,3′,4′-*penta*-*O*-methyl-8-(4-(diphenylamino)phenyl)quercetin (**10**) in 80% yield [21].

The demethylation of 8-phenyl-*penta*-*O*-methylquercetin (**8**) was investigated based on the reaction from **2** to **4** using BBr_3_ [20]. A slightly modified reaction condition using CPME as the solvent resulted in a high yield. As shown in Table 1, 3,7,3′,4′-*tetra*-*O*-methyl-8-phenylquercetin (**11**), 3,7,3′,4′-*tetra*-*O*-methyl-8-(1-naphthyl)quercetin (**12**), and 3,7,3′,4′-*tetra*-*O*-methyl-8-(4-(diphenylamino)-phenyl)quercetin (**13**) was obtained in 88%, 78%, and 87% yield, respectively. These results indicated that the reactivity of the 5-*O*-methyl group was the highest among the five methyl residues in **8**–**10**, as was found in **2**. As we reported [20], the subsequent demethylation reaction of the 3,7,3′,4′-*tetra*-*O*-methyl derivatives (**11**–**13**) was carried out using BCl_3_/TBAI (Table 2). These reaction conditions primarily yielded 3,7-*di*-*O*-methyl compounds. Demethylation of **11** afforded 3,7-*di*-*O*-methyl-8-phenylquercetin (**14**) in 85% yield. Using the same reaction conditions, 3,7-*di*-*O*-methyl-8-(1-naphthyl)quercetin (**15**) and 3,7-*di*-*O*-methyl-8-(4-(diphenylamino)phenyl)quercetin (**16**) were obtained in 62% and 82% yield, respectively.

### 2.2. Synthesis of O-Methylanthocyanidins

The as-prepared *O*-methylquercetins were transformed to cyanidin derivatives (Scheme 1). Our preliminary experiments suggested that Clemmensen-type reduction of 3,5,7,3′4′-*penta*-*O*-methylquercetin (**2**) using Zn/HCl [18] resulted in a messy product, and the yield of 3,5,7,3′4′-*penta*-*O*-methylcyanidin (**17**) was low. The well-known transformation from *penta*-*O*-methylquercetin (**2**) to 3,5,7,3′,4′-*penta*-*O*-methylcyanidin (**17**) using a combination of LiAlH_4_ and an oxidant was first reported in 1968 [34,35]. At that time, a large amount of LiAlH_4_ was used, and after the reduction, re-oxidation was carried out to afford anthocyanidins. We re-investigated the transformation reaction and found that the reduction of **2** with 4 eq. of LiAlH_4_ at room temperature afforded **17** in 70% yield without any oxidant [21]. In addition, quercetin derivatives possessing a 5-OH structure were transformed to the corresponding cyanidin compounds via a Clemmensen-type reduction [18]. Therefore, in this study, we employed both reaction conditions depending on the structure of the substrate.

The transformation from 3,7,3′,4′-*tetra*-*O*-methylquercetin (**4**) to 3,7,3′,4′-*tetra*-*O*-methylanthocyanidin (**18**) was investigated (Table 3). **4** and Zn powder were mixed in HCl-MeOH- tetrahydrofuran (THF) at 0 °C for 30 min under an Ar atmosphere, and then, the reaction mixture was exposed to air overnight after Zn removal to afford 3,7,3′,4′-*tetra*-*O*-methylcyanidin (**18**) as the only product in 83% yield. Transformation of **5**, **6**, and **14** using the same method afforded 3,7,4′-*tri*-*O*-methylcyanidin (**19**) and 3,7-*di*-*O*-methylcyanidin (**20**) and 3,7-*di*-*O*-methyl-8-phenylcyanidin (**24**) in 75%, 71%, and 38%, respectively (Table 3). It was demonstrated that our sequential reduction–oxidation anthocyanin synthesis using Zn could be applicable to *O*-methylquercetins. To prepare 8-aryl-3,5,7,3′,4′-*penta*-*O*-methylcyanidins (**21**–**23**) from 8-aryl-3,5,7,3′,4′-*penta*-*O*-methylquercetins (**8**–**10**), LiAlH_4_ reduction was applied [21]. **8** in THF was added to 4 equiv. of LiAlH_4_ and maintained at rt for 30 min. After purification by column chromatography, 8-phenyl-3,5,7,3′,4′-*penta*-*O*-methylcyanidin (**21**) was obtained in 41% yield. Using the same procedure, 3,5,7,3′,4′-*penta*-*O*-methyl-8-(1-naphthyl)cyanidin (**22**) and 3,5,7,3′,4′-*penta*-*O*-methyl-8-(4-(diphenylamino)phenyl)cyanidin (**23**) were prepared from the corresponding *O*-methylquercetins (**9** and **10**) in 57% and 66% yields, respectively.

### 2.3. Photovoltaic Property of DSSCs Using O-Methylquercetins

First, we fabricated DSSCs with five *O*-methylquercetins (**2**, **4**–**7**) where the number of methylations differed. Their photovoltaic properties with AM 1.5 irradiation are shown in Table 4. N719 was used as the standard dye. Although 4-*tert*-butylpyridine (TBP) was typically added to the electrolyte to increase *V*_oc_ [36,37], preliminary experiments indicated that TBP (0.5 mM) did not provide good results. Therefore, we performed the experiment without TBP. The cells with *penta*-*O*-methylquercetin (**2**), *tetra*-*O*-methylquercetin (**4**) and *tri*-*O*-methylquercetins (**5**) possessed a pale yellow color, and the cells with *di*-*O*-methylquercetin (**6**) and *mono*-*O*-methylquercetins (**7**) were orange, indicating that these dyes may be adsorbed to TiO_2_ with the dihydroxyl group of the B-ring. The efficiencies (*η*%) of the DSSCs of *O*-methylquercetins were not high (i.e., ranging from 0.09%–0.82%). Quercetin (**1**) and *mono*-*O*-methylquercetin (**7**) exhibited a similar efficiency (ca. 0.6%), and the highest (i.e., 0.82%) efficiency was observed for *di*-*O*-methylquercetin (**6**). We proposed that methylation may increase the hydrophobicity of the compounds, which would result in an increase of the efficiency. However, this behavior was not observed. The incident photon-to-current conversion efficiency (IPCE) spectra indicated that these compounds only absorbed light energy shorter than 500 nm, and a peak was observed at approximately 400 nm at a maximum of 60% with *di*-*O*-methylquercetin (**6**).

Because *O*-methylquercetins are flat molecules that most likely stack on each other followed by quenching the harvested energy via intermolecular charge transfer, a hindered residue was introduced to prevent this phenomenon. In addition, an aryl group at the 8-position of the quercetin chromophore was introduced as an electron-donating residue, which may increase HOMO level. Based on results from preliminary experiments, we chose the 8-phenyl, 8-naphthyl and 8-(4-(diphenylamino)phenyl-residues for the substituents and prepared nine 8-aryl-*O*-methylquercetins (**8**–**16**) for use as DSSC dyes. The photovoltaic properties are shown in Table 5. Similar to the methylquercetins, the cell color of the *penta*-*O*-methyl derivatives and *tetra*-*O*-methyl derivatives were yellow, and the *di*-*O*-methyl compounds were orange. Among these compounds, the efficiency of 8-naphthyl-3,7,3′,4′-*tetra*-*O*-methylquercetin (**12**) exhibited a high value of 1.04% (Table 5). Other 8-aryl-3,7,3′,4′-*tetra*-*O*-methylquercetins also exhibited a higher *η*% compared to that of *tetra*-*O*-methylquercetin (**4**). The efficiencies of the 8-aryl-3,7-*di*-*O*-methylquercetins (**14**–**16**) were 0.67%–0.88%, which is nearly the same as that of 3,7-*di*-*O*-methylquercetins (**6**). The introduction of the 8-aryl residue to 3,5,7,3′,4′-*penta*-*O*-methylquercetin did not result in any increase in *η*% and exhibited a very low efficiency of 0.03%–0.06%. For these nine compounds, the efficiency may be affected by a balance of many factors, such as the hydrophobicity, electron density, and the adsorption to TiO_2_, which may cause complicated results.

### 2.4. Photovoltaic Property of DSSCs Using O-Methylcyanidins

Using metal reduction and hydride reagent reduction, *O*-methylcyanidins (**17**–**24**) were prepared, and DSSCs were fabricated. Then, their photovoltaic properties were measured (Table 6). The cells of the DSSCs fabricated with *penta*- (**17**), *tetra*- (**18**) and *tri*-*O*-methylcyanidins (**19**), 8-aryl-3,5,7,3′,4′-*penta*-*O*-methylcyanidins (**21**–**23**) were red, and the cell of the DSSCs fabricated with *di*-*O*-methylcyanidin (**20**) and 8-phenyl-3,7-*di*-*O*-methylcyanidin (**24**) were blue. The red color dyes may exist without a tight chemical adsorption of TiO_2_. In the case of the blue color dyes, they might be due to the bond formation between the catechol-type B-ring and Ti atoms in TiO_2_ [28].

The efficiencies (*η*%) of the DSSCs of *O*-methylcyanidins (**17**–**19**, **21**–**23**) without the catechol moiety were quite low and ranged from 0.08% to 0.22%. These low efficiencies (*η*%) could be attributed to the fact that the adsorption amount might be low because of a lack of di-OH at B-ring chromophore. On the other hand, *di*-*O*-methylcyanidins (**20**, **24**) with the catechol moiety exhibited a higher value of *η*% (0.32% for **20**, 0.52% for **24**). This increase might be due to the bond formation between the catechol moiety and TiO_2_. Because the efficiency (*η*%) of 8-phenyl-3,7-*di*-*O*-methylcyanidin (**24**) was higher than that of 3,7-*di*-*O*-methylcyanidin (**20**), a substitution effect at the 8-position was confirmed. However, the efficiency (*η*% = 0.52) of **24** was lower than that (*η*% = 0.67) of the 8-phenyl-*di*-*O*-methylquercetin (**14**). These results indicated that the introduction of the electron-donating aryl group at the 8-position increased the efficiency (*η*%) of the DSSCs when the dyes bonded to TiO_2_.

## 3. Materials and Methods

### 3.1. General

The melting points were measured on a Yanaco Mp-S3 micro melting point apparatus and are uncorrected. The infrared (IR) spectra were recorded on a JASCO FT/IR-460Plus spectrometer, and the ultraviolet-visible (UV-Vis) absorption spectra were recorded on a JASCO V-560 spectrophotometer (JASCO, Sapporo, Japan, cell length: 10 mm). The ^1^H and ^13^C nuclear magnetic resonance (NMR) spectra were recorded on a JEOL JNM-ECA-500 spectrometer (JEOL, Tokyo, Japan). The chemical shifts are reported in ppm on the δ scale relative to CDCl_3_ (δ = 7.26 for ^1^H-NMR), CDCl_3_ (δ = 77.0 for ^13^C-NMR), CD_3_OD (δ = 3.31 for ^1^H-NMR), CD_3_OD (δ = 49.0 for ^13^C-NMR), DMSO-*d_6_* (δ = 2.49 for ^1^H-NMR), and DMSO-*d_6_* (δ = 39.5 for ^13^C-NMR) as internal references. The signal patterns are as follows: s, singlet; brs, broad singlet; d, doublet; brd, broad doublet; t, triplet; m, multiplet. High-resolution mass spectra (HRMS) were recorded on a Bruker micrOTOF-QII (ESI) spectrometer. Analytical thin-layer chromatography (TLC) was performed on Merck precoated analytical plates (0.25 mm thick, silica gel 60 F254, MERCK MILLIPORE, Darmstadt, Germany). High-performance liquid chromatography (HPLC) was performed using a system equipped with JASCO PU-1580 pumps, an MD-1515 detector, HG-1580-32 mixer, and DG-580-35 degasser (JASCO). An Octadecylsilane (ODS) column (SunShell C18, 2.6 µm, 2.1 inside diameter × 100 mm, ChromaNik Technologies, Osaka, Japan) was eluted at 40 °C using linear gradient elution (0.2 mL/min) over 20 min from 10% to 90% aq. MeCN solution containing 0.5% trifluoroacetic acid (TFA). Flash silica gel chromatography was performed using silica gel PSQ60B (Fuji Silysia Chemical, Kasugai, Japan). All reagents were purchased from commercial sources. All reactions were performed under an argon (Ar) atmosphere unless otherwise noted.

To coat the TiO_2_ paste (Solaronix Ti-Nanoxide, Aubonne, Switzerland) onto fluorine-doped tin oxide (FTO) glass plates (Astellatech Co., Ltd., Yokohama, Japan, 75 mm × 25 mm × 1.8 mm thick), screen-printing equipment (WHT3, Mino International, Ltd., Tokyo, Japan and HP-320, Newlong Seimitsu Kogyo Co., Ltd., Tokyo, Japan) was used. Calcination of the TiO_2_-coated FTO glass was performed using a furnace (FT-101W, Full-Tech Co., Ltd., Osaka, Japan). N719 (Aldrich, Tokyo, Japan, 95%) was used as the standard dye.

### 3.2. Synthesis of O-Methylquercetins

#### 3.2.1. 3,7,3′,4′-*tetra*-*O*-Methyl-8-phenylquercetin (**11**)

To a solution of **8** (710 mg, 1.6 mmol) in cyclopentyl methyl ether (CPME) (20 mL) was added BBr_3_ (4 mL, 4.0 mmol, 1.0 M solution in CH_2_Cl_2_) dropwise at 0 °C. After stirring for 5 min at 0 °C and for 60 min at rt, MeOH (25 mL) was poured into the mixture. The reaction mixture was concentrated under reduced pressure and saturated aq. NaCl (30 mL) was added. After the mixture was extracted with CH_2_Cl_2_ (15 mL × 3), the combined organic phases were washed with saturated aq. NaCl (30 mL), dried over anhydrous Na_2_SO_4_ and concentrated under reduced pressure. The crude product was recrystallized from AcOEt (20 mL) to give **11** as a yellow solid (607 mg, 88%). mp: 225–226 °C; IR (KBr) cm^−1^; 2995, 1585, 1517, 1267, 1217 cm^−1^; UV-Vis (CDCl_3_) λ_max_ nm (ε): 276 nm (20,160), 366 nm (26,960); ^1^H-NMR (CDCl_3_, 500 MHz) δ 3.61 (3H, s), 3.84 (3H, s), 3.90 (3H, s), 3.91 (3H, s), 6.50 (1H, s), 6.86 (1H, d, *J* = 9.0 Hz), 7.17 (1H, d, *J* = 2.5 Hz), 7.3–7.4 (3H, m), 7.4–7.5 (2H, m), 7.76 (1H, dd, *J* = 9.0, 2.5 Hz); ^13^C-NMR (CDCl_3_, 125 MHz) δ 55.8, 55.9, 56.2, 60.0, 94.9, 105.4, 109.5, 110.3, 110.8, 122.7, 123.0, 127.3, 128.1, 131.2, 132.2, 138.8, 148.7, 151.2, 152.8, 155.3, 161.7, 162.3, 179.1; ESI-TOF HRMS Calcd. For C_25_H_23_O_7_ [M + H]^+^, 435.1438; Found: 435.1440.

#### 3.2.2. 3,7,3′,4′-*tetra*-*O*-Methyl-8-(1-naphthyl)quercetin (**12**)

According to the procedure described for **11**, the reaction of **9** (400 mg, 0.80 mmol) was carried out to afford **12** (304 mg, 78%) as a yellow solid. mp: 239–240 °C; IR (KBr) cm^−1^; 3437, 2935, 1648, 1589, 1218 cm^−1^; UV-Vis (CDCl_3_) λ_max_ nm (ε): 275 nm (27,800), 364 nm (20,940); ^1^H-NMR (CDCl_3_, 500 MHz) δ 3.16 (3H, s), 3.76 (3H, s), 3.83 (3H, s), 3.90 (3H, s), 6.58 (1H, s), 6.59 (1H, d, *J* = 2.5 Hz), 6.72 (1H, d, *J* = 8.5 Hz), 7.3–7.4 (1 H, m), 7.4–7.5 (2H, m), 7.5–7.6 (3H, m), 7.8–7.9 (2H, m); ^13^C-NMR (CDCl_3_, 125 MHz) δ 55.2, 55.8, 56.2, 59.9, 95.0, 105.5, 107.2, 109.7, 110.6, 122.6, 122.8, 125.6, 125.9, 126.3, 128.1, 128.2, 129.0, 148.5, 151.0, 153.4, 155.4, 162.2, 163.1, 179.1; ESI-TOF HRMS Calcd. For C_29_H_25_O_7_ [M + H]^+^, 485.1595; Found: 485.1592.

#### 3.2.3. 3,7,3′,4′-*tetra*-*O*-Methyl-8-(4-(diphenylamino)phenyl)quercetin (**13**)

According to the procedure described for **11**, the reaction of **10** (210 mg, 0.34 mmol) was carried out to afford **13** (179 mg, 87%) as a yellow solid. mp: 260–261 °C; IR (KBr) cm^−1^; 3435, 2992, 1587, 1488, 1266 cm^−1^; UV-Vis (CDCl_3_) λ_max_ nm (ε): 254 nm (24,060), 312 nm (39,080), 363 nm (16,600); ^1^H-NMR (CDCl_3_, 500 MHz) δ 3.80 (3H, s), 3.87 (3H, s), 3.90 (3H, s), 3.95 (3H, s), 6.50 (1H, s), 6.87 (1H, d, *J* = 9.0 Hz), 7.0–7.3 (14H, m), 7.54 (1H, d, *J* = 2.0 Hz), 7.64 (1H, dd, *J* = 9.0, 2.0 Hz); ^13^C-NMR (CDCl_3_, 125 MHz) δ 56.0, 56.2, 56.3, 60.1, 95.0, 105.5, 109.1, 110.7, 111.1, 122.5, 122.6, 123.1, 124.6, 125.5, 129.3, 131.9, 138.7, 146.8, 147.6, 148.7, 151.3, 153.0, 155.6, 161.5, 162.4, 179.1; ESI-TOF HRMS Calcd. For C_37_H_32_NO_7_ [M+H]^+^, 602.2173; Found: 602.2177.

#### 3.2.4. 3,7-*di*-*O*-Methyl-8-phenylquercetin (**14**)

To a solution of **11** (150 mg, 0.35 mmol) and tetrabutylammonium iodide (TBAI) (0.68 g, 1.83 mmol) in CH_2_Cl_2_ (3 mL) was added BCl_3_ (1.83 mL, 1.83 mmol, 1.0 M solution in CH_2_Cl_2_) dropwise at −20 °C. After stirring for 60 min at −20 °C, MeOH (4.5 mL) was poured into the mixture. The reaction mixture was concentrated under reduced pressure and saturated aq. Na_2_S_2_O_3_ (20 mL) was added. After the mixture was extracted with AcOEt (15 mL × 3), the combined organic phases were washed with saturated aq. NaCl (20 mL), dried over anhydrous Na_2_SO_4_ and concentrated under reduced pressure. The residue was purified by flash silica gel chromatography (*n*-hexane/AcOEt = 1:2 to AcOEt) to give **14** as a yellow solid (119 mg, 85%). mp: 261–262 °C; IR (KBr) cm^−1^; 3476, 1649, 1595, 1442, 1274 cm^−1^; UV-Vis (MeOH) λ_max_ nm (ε): 258 nm (35,800), 372 nm (23,800); ^1^H-NMR (DMSO-*d_6_*, 500 MHz) δ 3.80 (3H, s), 3.82 (3H, s), 6.66 (1H, s), 6.70 (1H, d, *J* = 8.5 Hz), 7.01 (1H, dd, *J* = 8.5, 2.5 Hz), 7.4–7.5 (6H, m), 9.19 (OH, brs), 9.80 (OH, brs); ^13^C-NMR (DMSO-*d_6_*, 125 MHz) δ 56.4, 59.6, 95.2, 104.6, 108.9, 115.3, 115.9, 120.8, 127.3, 127.9, 131.1, 131.4, 137.5, 145.1, 148.7, 152.2, 156.0, 160.8, 161.9, 178.4; ESI-TOF HRMS Calcd. For C_23_H_19_O_7_ [M + H]^+^, 407.1125; Found: 407.1133.

#### 3.2.5. 3,7-*di*-*O*-Methyl-8-(1-naphthyl)quercetin (**15**)

According to the procedure described for **14**, the reaction of **12** (150 mg, 0.31 mmol) was carried out to afford **15** (88 mg, 62%) as a yellow solid. mp: 309–310 °C; IR (KBr) cm^−1^; 3479, 3152, 1601, 1438, 1207 cm^−1^; UV-Vis (MeOH) λ_max_ nm (ε): 258 nm (41,060), 371 nm (29,600); ^1^H-NMR (DMSO-*d_6_*, 500 MHz) δ 3.76 (3H, s), 3.80 (3H, s), 6.4–6.5 (2H, m), 6.75 (1H, s), 7.16 (1H, d, *J* = 2.0 Hz), 7.3–7.6 (6H, m), 8.0–8.1 (2H, m), 9.05 (OH, brs), 9.72 (OH, brs), 13.03 (OH, brs); ^13^C-NMR (DMSO-*d_6_*, 125 MHz) δ 56.4, 59.6, 95.2, 104.8, 106.7, 115.1, 115.7, 119.6, 120.1, 125.2, 125.5, 125.7, 126.2, 128.0, 128.2, 129.1, 132.8, 137.4, 144.9, 148.6, 152.8, 155.7, 161.3, 162.6, 178.4; ESI-TOF HRMS Calcd. For C_27_H_21_O_7_ [M + H]^+^, 457.1282; Found: 457.1280.

#### 3.2.6. 3,7-*di*-*O*-Methyl-8-(4-(diphenylamino)phenyl)quercetin (**16**)

According to the procedure described for **14**, the reaction of **13** (150 mg, 0.25 mmol) was carried out to afford **16** (117 mg, 82%) as a yellow solid. mp: 271–272 °C; IR (KBr) cm^−1^; 3434, 1645, 1592, 1489, 1206 cm^−1^; UV-Vis (MeOH) λ_max_ nm (ε): 257 nm (14,600), 304 nm (22,800), 368 nm (10,000); ^1^H-NMR (DMSO-*d_6_*, 500 MHz) δ 3.80 (3H, s), 3.83 (3H, s), 6.63 (1H, s), 6.76 (1H, d, *J* = 8.0 Hz), 7.0–7.4 (15H, m), 7.47 (1H, d, *J* = 2.0 Hz), 9.24 (OH, brs), 9.91 (OH, brs), 13.0 (OH, brs); ^13^C-NMR (DMSO-*d_6_*, 125 MHz) δ 56.5, 59.6, 95.1, 104.7, 108.5, 115.3, 116.0, 120.2, 120.9, 122.8, 123.0, 123.9, 125.6, 129.6, 132.1, 137.5, 145.2, 146.3, 147.2, 148.8, 152.4, 155.8, 160.6, 178.4; ESI-TOF HRMS Calcd. For C_35_H_28_NO_7_ [M + H]^+^, 574.1860; Found: 574.1849.

### 3.3. Synthesis of O-Methylanthocyanidins

#### 3.3.1. 3,7,3′,4′-*tetra*-*O*-Methylcyanidin (**18**)

To a suspension of **4** (200 mg, 0.56 mmol) and Zn powder (2.0 g, 31 mmol) in anhydrous MeOH (8.0 mL) and anhydrous THF (2.0 mL) was added 4 N hydrogen chloride**-**methanol solution (10 mL) at 0 °C. After stirring for 30 min at 0 °C, Zn was removed by suction filtration. The filtrate solution was stirred under air for 20 h. After the reaction solution was added with water containing 0.5% trifluoroacetic acid (TFA) (100 mL), MeOH and THF were removed under reduced pressure. The residual aqueous solution was extracted with CH_2_Cl_2_ (20 mL × 4). The combined organic phases were dried over anhydrous Na_2_SO_4_ and concentrated under reduced pressure. The residue was purified by flash silica gel chromatography (33% AcOEt/Hexane with 0.5% TFA → AcOEt with 0.5% TFA → 5% MeOH/AcOEt with 0.5% TFA) to give **18** as a TFA salt (dark red) solid (210 mg, 83%). mp: 110–111 °C; IR (KBr) cm^−1^; 2935, 1613, 1589, 1319, 1209 cm^−1^; UV-Vis (0.1%HCl-MeOH) λ_max_ nm (ε): 279 nm (18,000), 522 nm (26,600); ^1^H-NMR (10%TFA-*d*-CDCl_3_, 500 MHz) δ 4.02 (3H,s), 4.05 (3H, s), 4.08 (3H,s), 4.23 (3H,s), 6.84 (1H, s), 6.95 (1H, s), 7.14 (1H, d, *J* = 9.0 Hz), 7.97 (1H, d, *J* = 2.5 Hz), 8.37 (1H, dd, *J* = 9.0, 2.5 Hz), 8.79 (1H, s), ^13^C-NMR (10%TFA-*d*-CDCl_3_, 125 MHz) δ 56.4, 56.6, 56.8, 57.8, 92.5, 103.3, 112.0, 112.8, 112.9, 120.9, 128.8, 130.3, 147.9, 149.4, 155.3, 155.6, 156.6, 162.6, 169.2; ESI-TOF HRMS Calcd. For C_19_H_19_O_6_ [M]^+^, 343.1176; Found: 343.1170.

#### 3.3.2. 3,7,4′-*tri*-*O*-Methylcyanidin (**19**)

According to the procedure described for **18**, the reaction of **5** (200 mg, 0.58 mmol) was carried out to afford **19** (192 mg, 75%) as a TFA salt (dark red) solid. mp: 144–145 °C; IR (KBr) cm^−1^; 3284, 1680, 1574, 1375, 1204 cm^−1^; UV-Vis (0.1%HCl-MeOH) λ_max_ nm (ε): 280 nm (11,080), 525 nm (17,320); ^1^H-NMR (10%TFA-*d*-CD_3_OD, 500 MHz) δ 3.95 (3H, s), 3.98 (3H,s), 4.17 (3H,s), 6.66 (1H, d, *J* = 2.0 Hz), 7.02 (1H, d, *J* = 2.0 Hz), 7.03 (1H, d, *J* = 9.0 Hz), 7.89 (1H, d, *J* = 2.0 Hz), 8.14 (1H, dd, *J* = 9.0, 2.0 Hz), 8.70 (1H, s), ^13^C-NMR (10%TFA-*d*-CD_3_OD, 125 MHz) δ 56.9, 57.5, 58.3, 92.7, 103.3, 112.9, 114.3, 117.8, 122.4, 127.9, 130.8, 148.6, 149.4, 156.7, 157.8, 163.8, 170.0; ESI-TOF HRMS Calcd. For C_18_H_17_O_6_ [M]^+^, 329.1020; Found: 329.1023.

#### 3.3.3. 3,7-*di*-*O*-methylcyanidin (**20**).

According to the procedure described for **18**, the reaction of **6** (200 mg, 0.61 mmol) was carried out to afford **20** (183 mg, 71%) as a TFA salt (dark red) solid. mp: 121–122 °C; IR (KBr) cm^−1^; 3419, 1682, 1455, 1210, 1128 cm^−1^; UV-Vis (0.1%HCl-MeOH) λ_max_ nm (ε): 280 nm (9600), 527 nm (11,600); ^1^H-NMR (10%TFA-*d*-CD_3_OD, 500 MHz) δ 3.98 (3H, s), 4.18 (3H,s), 6.67 (1H, d, *J* = 2.0 Hz), 6.98 (1H, d, *J* = 9.0 Hz), 7.05 (1H, d, *J* = 2.0Hz), 8.04 (1H, d, *J* = 2.5 Hz), 8.15 (1H, dd, *J* = 9.0, 2.5 Hz), 8.68 (1H, s), ^13^C-NMR (10%TFA-*d*-CD_3_OD, 125 MHz) δ 57.4, 58.2, 92.6, 103.1, 113.6, 117.6, 118.7, 121.3, 128.6, 129.7, 147.6, 149.3, 156.6, 157.7, 164.5, 169.5; ESI-TOF HRMS Calcd. For C_17_H_15_O_6_ [M]^+^, 315.0863; Found: 315.0864.

#### 3.3.4. 3,7-*di*-*O*-Methyl-8-phenylcyanidin (**24**)

According to the procedure described for **18**, the reaction of **14** (80 mg, 0.61 mmol) was carried out to afford **24** (38 mg, 38%) as a TFA salt (dark red) solid. mp: 250–251 °C; IR (KBr) cm^−1^; 3408, 3107, 1679, 1207, 1142 cm^−1^; UV-Vis (0.1%HCl-MeOH) λ_max_ nm (ε): 281 nm (7700), 538 nm (7100); ^1^H-NMR (10%TFA-*d*-CD_3_OD, 500 MHz) δ 3.98 (3H, s), 4.22 (3H,s), 6.82 (1H, d, *J* = 8.5 Hz), 6.99 (1H, s), 7.4–7.6 (5H, m), 7.69 (1H, dd, *J* = 8.5, 2.0 Hz), 7.85 (1H, d, *J* = 2.0 Hz), 8.80 (1H, s), ^13^C-NMR (10%TFA-*d*-CD_3_OD, 125 MHz) δ 57.5, 58.2, 92.3, 98.9, 111.5, 117.3, 119.6, 121.5, 128.2, 129.4, 129.6, 129.9, 131.6, 132.1, 147.4, 149.1, 165.8, 171.8; ESI-TOF HRMS Calcd. For C_23_H_19_O_6_ [M]^+^, 391.1176; Found: 391.1181.

### 3.4. Preparation of DSSCs

We prepared the DSSCs according to the method reported by Liu et al. with slight modifications [28,38]. TiO_2_ films for use as photoanodes were prepared by screen-printing TiO_2_ pastes with different particle diameters (11–400 nm) onto F-doped SnO_2_ glass. After treating the glass with a TiCl_4_ solution, the films were calcined using the following temperature program: heat from room temperature (rt) to 200 °C for 15 min, from 200 to 500 °C for 15 min, hold at 500 °C for 30 min, and then cool to rt. The TiO_2_ electrodes were immersed in methanol or acetonitrile solution and then maintained at room temperature (rt) for or 18 h. After dye loading, the films were removed and rinsed with acetonitrile. The dye-loaded TiO_2_ electrodes were sandwiched between commercially available Pt counter electrodes (Geomatec Co., Ltd., Yokohama, Japan) with electrolyte filling the gap separated by a spacer (HIMILAN: DuPont-Mitsui Polychemicals Co., Ltd., Tokyo, Japan). The active area of the cells was 0.16 cm^2^. Iodide (I^−^/I_3_^−^), which is a commonly used electrolyte, was used as the electrolyte in this study [38]. 4-*tert*-Butylpyridine (TBP) was occasionally added. The thicknesses of the TiO_2_ films on the FTO glasses were measured after the performance evaluation using a SURFCOM 130A (ACCRETECH Co., Ltd., Tokyo, Japan). The average thickness was approximately 10 ± 1 µm.

### 3.5. Measurement of the Cell Properties

The measurement of the cell properties was performed according to a previously reported protocol [28,38]. The current–voltage (*J*–*V*) characteristics of the cells were measured using an AM 1.5 solar simulator (OTENTO-SUN III, Bunkoukeiki Co., Ltd., Tokyo, Japan). The data were collected by a source meter (Keithley 2400), and the light-to-electricity conversion efficiency (*η*) was obtained from the following equation: *η* = (*J*_sc_ × *V*_oc_ × *FF*)/*P*_in_, where *J*_sc_ is the short-circuit photocurrent density, *V*_oc_ is the open-circuit voltage, *FF* is the fill factor, and *P*_in_ is the incident radiation power. The incident monochromatic photon-to-current conversion efficiency (IPCE) spectra were measured using an IPCE measurement system (SM-250 hyper mono light system, Bunkoukeiki Co., Ltd.). The IPCE values were obtained by comparing the current ratio and the IPCE value of the reference cell at each wavelength. The light intensity of the illumination source was adjusted using standard silicon photodiodes, i.e., BS520 for *J*–*V* characteristics and SiPD S1337-1010BQ for external quantum efficiency (EQE) measurements (Bunkoukeiki Co., Ltd.).

## 4. Conclusions

We designed and synthesized four *O*-methylcyanidins, as well as three types of 8-aryl-*O*-methylquercetins and cyanidins, including several new compounds. The introduction of the 8-aryl residue was established with a Suzuki-Miyaura coupling of 8-iodo-*per*-*O*-methylquercetin, and reduction of the quercetin derivatives to the corresponding cyanidin compounds was achieved in satisfactory yield. This synthetic route is very practical and efficient for the preparation of substituted flavonols and anthocyanidins. In the near future, the function of these compounds to prevent metabolic syndromes in humans will be studied. Using these flavonoids, we fabricated DSSCs and evaluated their photovoltaic efficiency. The efficiency was relatively low compared to that of natural flavonols and anthocyanins, which may be due to intermolecular stacking of the compounds, and the harvested energy may be quickly quenched before the electron reaches the electrode. Further molecular design and preparations are underway.

## Figures and Tables

**Table 1:** Synthesis of 3,7,3′,4′-*tetra*-*O*-methylquercetins (**11**–**13**).

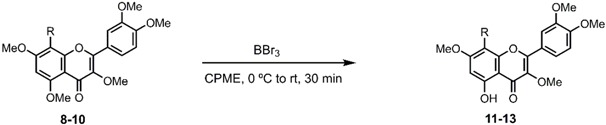			
*penta*-*O*-Methylquercetin	R	*tetra*-*O*-Methylquercetin	Yield (%) ^a^
8	phenyl	11	88
9	1-naphthyl	12	78
10	4-(diphenylamino)phenyl	13	87

^a^ Isolated yield.

**Table 2:** Synthesis of 3,7-*di*-*O*-methylquercetins (**14**–**16**).

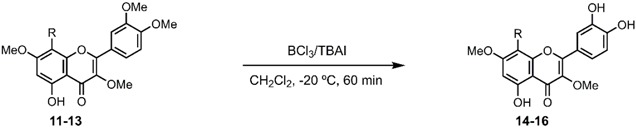			
*tetra*-*O*-Methylquercetin	R	*di*-*O*-Methylquercetin	Yield (%) ^a^
11	phenyl	14	85
12	1-naphthyl	15	62
13	4-(diphenylamino)phenyl	16	82

^a^ Isolated yield.

**Table 3:** Synthesis of *O*-methylanthocyanidins (**18**–**20**, **24**) using Zn. ^a^ Isolated yield.

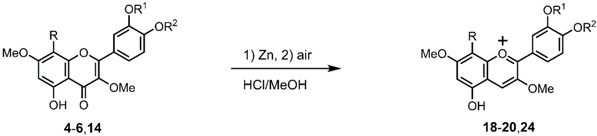					
*O*-Methylquercetin	R	R^1^	R^2^	*O*-Methylanthocyanidin	Yield (%) ^a^
4	H	Me	Me	18	83
5	H	H	Me	19	75
6	H	H	H	20	71
14	phenyl	H	H	24	38

**Table 4:** Photovoltaic properties of the dye-sensitized solar cells (DSSCs) sensitized with *O*-methyl quercetins (**1**, **2**, **4**–**7**) ^a,b^.

Dye	Solvent	*J*_sc_ (mA/cm^2^)	*V*_oc_ (mV)	*FF*	*η* (%)
*penta*-OMeQ (**2**)	MeOH	0.45	390	0.56	0.09
*tetra*-OMeQ (**4**)	MeOH	1.17	450	0.59	0.31
*tri*-OMeQ (**5**)	MeOH	2.04	439	0.60	0.54
*di*-OMeQ (**6**)	MeOH	4.65	445	0.40	0.82
*mono*-OMeQ (**7**)	MeOH	3.60	424	0.40	0.62
quercetin (**1**)	MeOH	3.09	459	0.43	0.60
N719	MeCN/*tert*-BuOH	15.7	717	0.64	7.3

^a^ Dyes were dissolved in MeOH with the concentration of 0.5 mM and immersed for 3 h; ^b^ For measurement of photovoltaic properties I^−^/I_3_^−^ was used as an electrolyte.

**Table 5:** Photovoltaic properties of the DSSCs sensitized with 8-aryl-*O*-methylquercetins (**8**–**16**) ^a,b^.

Dye	Solvent	*J*_sc_ (mA/cm^2^)	*V*_oc_ (mV)	*FF*	*η* (%)
8-aryl-*penta*-OMeQ					
8-phenyl (**8**)	MeOH	0.21	257	0.59	0.03
8-naphthyl (**9**)	MeOH	0.25	264	0.62	0.04
8-(4-(diphenylamino)phenyl) (**10**)	MeOH	0.28	350	0.59	0.06
8-aryl-*tetra*-OMeQ					
8-phenyl (**11**)	CH_2_Cl_2_	2.15	486	0.63	0.66
8-naphthyl (**12**)	MeOH ^c^	2.35	663	0.70	1.04
8-(4-(diphenylamino)phenyl) (**13**)	CH_2_Cl_2_	2.96	535	0.55	0.87
8-aryl-*di*-OMeQ					
8-phenyl (**14**)	MeOH	3.73	451	0.40	0.67
8-naphthyl (**15**)	MeOH	4.55	436	0.44	0.88
8-(4-(diphenylamino)phenyl) (**16**)	MeOH	4.75	345	0.47	0.77

^a^ Dyes were dissolved in each solvent with the concentration of 0.5 mM and immersed for 3 h; ^b^ For measurement of photovoltaic properties I^−^/I_3_^−^ was used as an electrolyte; ^c^ TBP (4-*tert*-butylpyridine) was added to the electrolyte.

**Table 6:** Photovoltaic properties of the DSSCs sensitized with *O*-methylcyanidins (**17**–**24**) ^a,b^.

Dye	*J*_sc_ (mA/cm^2^)	*V*_oc_ (mV)	*FF*	*η* (%)
*penta*-OMeCy (**17**)	0.55	281	0.50	0.08
*tetra*-OMeCy (**18**)	0.71	284	0.63	0.13
*tri*-OMeCy (**19**)	0.51	266	0.63	0.08
*di*-OMeCy (**20**)	1.41	356	0.64	0.32
8-phenyl-*penta*-OMeCy (**21**)	1.18	297	0.64	0.22
8-naphthyl-*penta*-OMeCy (**22**)	0.58	346	0.60	0.12
8-(4-(diphenylamino)phenyl)-*penta*-OMeCy (**23**)	0.29	272	0.59	0.05
8-phenyl-*di*-OMeCy (**24**)	2.43	328	0.65	0.52

^a^ Dyes were dissolved in MeCN with the concentration of 0.5 mM and immersed for 14 h; ^b^ For measurement of photovoltaic properties I^−^/I_3_^−^ was used as an electrolyte.
